# Nutritive Properties of Coffee

**Published:** 1850-09

**Authors:** 


### MATERIA MEDICA AND THERAPEUTICS.

*Nutritive Properties of Coffee.*—M. Gasparin stated, a short time ago, before the Academy of Sciences of Paris, that the journeymen miners in the neighborhood of Charleroi preserve robust health and great muscular strength by using an article of food containing but half the nutritive principles which are found in the usual nourishment used in the rest of Europe among people occupied in the same manner. The difference lies principally in the large quantities of coffee which the Charleroi miners consume. The journeyman, before going to his work in the morning, takes half a pint of coffee (half chicory and half coffee) and a good slice of bread-and-butter. He carries with him into the mine another pint of coffee and a few rounds of bread-and-butter; and after returning home in the evening, he sups upon potatoes and cabbage, or some other green vegetable, and concludes his meal with a repetition of coffee and bread-and-butter. The consumption per diem consists in about two pounds of bread, two ounces of butter, one ounce of coffee, and another of chicory, and a pound and a half of cooked vegetables. Meat is eaten on Sundays and holidays only, and the miner drinks no fermented liquors during the week, his only beverage being his coffee. On Sundays, however, he takes a couple of quarts of beer. Thus it is calculated that such a laborer consumes only about four drachms of nitrogen per diem, whilst in other places about double the quantity is required. It would appear from Boecker's investigations, (see *The Lancet,* vol. i. 1850, p. 119,) that the amount of urea ejected in the twenty-four hours is diminished to one-half by the use of coffee. This substance would, then, seem to prevent the loss of nitrogen, and thus its nutritive effects might be explained. The garlic-bulbs, so much used in the south of France, seem to act in a similar manner. It has, on the other hand, been ascertained by M. Barral, that common salt increases the proportion of urea and uric acid, so that more substantial nourishment is required by those who use table salt. Government might very judiciously, therefore, favor the use of coffee among the humbler classes, and free it from all duty. It would be interesting to institute a few inquiries regarding the effects produced in this country by the use of tea.—*Ibid.*

